# Breast adenocarcinoma recurring as small cell carcinoma in a patient with a germline *BRCA2* mutation: clonal evolution unchecked

**DOI:** 10.1186/2162-3619-4-1

**Published:** 2015-01-06

**Authors:** Polly Niravath, Tanya Eble, Alejandro Contreras, Marilyn Li, Luis M Franco, Mothaffar Rimawi

**Affiliations:** Lester & Sue Smith Breast Center, Baylor College of Medicine, One Baylor Plaza, BCM 660, Houston, Texas 77030 USA; Department of Molecular and Human Genetics, Baylor College of Medicine, One Baylor Plaza, BCM 320, Houston, TX 77030 USA; Department of Pathology, Baylor College of Medicine, One Baylor Plaza, BCM 660, Houston, TX 77030 USA

**Keywords:** *BRCA2* mutation, Small cell carcinoma of the breast, *PIK3CA* mutation, Therapeutic stress

## Abstract

**Background:**

Because up to 30% of breast cancer cases may relapse, understanding the biology of recurrent breast cancer is imperative in preventing these poor outcomes. Thus, we present this unusual case of a BRCA2 carrier who presented seven years after her initial diagnosis of breast adenocarcinoma with a new lump in the left axillary tail, which proved to be small cell carcinoma. The second cancer bore no morphologic or immunohistochemical resemblance to the first. However, we aimed to understand whether the two cancers could have been related.

**Methods:**

We performed targeted Next Generation Sequencing on both cancer specimens in order to determine whether there was a genomic relationship between the two cancers.

**Results:**

We found that the initial breast adenocarcinoma was positive for a heterozygous mutation in *PIK3CA* (c. 1624 G > A, p.E42K) and a heterozygous 13-basepair deletion in TP53 (c.639-651del, p.H214fs). The small cell cancer was positive for the same mutation in *PIK3CA*, but negative for the mutation in *TP53.*

**Conclusion:**

We concluded that the small cell cancer may have arisen from a clone within the initial cancer, since they carried an identical genetic mutation. Furthermore, we postulate that the unusual morphology of the second cancer may be due, in part, to the patient’s germline BRCA mutation.

## Background

Small cell carcinoma of the breast is a very rare cancer, with fewer than 30 cases reported in the literature [1]. It is not clear how primary small cell cancer of the breast occurs, since neuroendocrine cells are not found in normal breast tissue. However, extra-pulmonary small cell cancers can form in the ovary and prostate, which also do not contain neuroendocrine cells. Several case series report an in situ component of the small cell carcinoma within the breast, indicating that the cancer can potentially begin in the breast [1, 2]. Some case series also describe tumors with a dimorphic appearance, with small cell cancer juxtaposed with invasive lobular cancer, or typical ductal cancer [2–4]. Interestingly, there is one report of a small cell tumor and an adjacent intraductal cancer (DCIS), which showed identical loss of heterozygosity (LOH) in 11 allelic loci in the DCIS and the small cell carcinoma, suggesting that the small cell carcinoma may have been clonally related to the DCIS [4].

Because many of these tumors are poorly differentiated, immunohistochemistry is not always consistent; diagnosis should rest primarily on morphology rather than immunohistochemical stains. However, small cell carcinomas of the breast may express cytokeratin 7 (CK7), CAM 5.2, neuron specific enolase (NSE), chromogranin, and synaptophysin. They are typically negative for cytokeratin 20. There are some reports of estrogen and progesterone positivity. However, HER2-positive small cell carcinomas of the breast are extremely rare [2]. Up to 53% of extra-pulmonary small cell carcinomas are known to express TTF-1 as well [5].

In this case, we discuss a *BRCA2* mutation carrier who initially presented with invasive ductal carcinoma of the left breast, and then developed a small cell carcinoma of the left breast several years after her initial treatment was completed. We discuss the phylogeny of this small cell breast cancer, and whether it may have been a very distorted recurrence of the initial breast cancer in this patient with a known BRCA2 mutation.

## Case presentation

**Figure 1:**
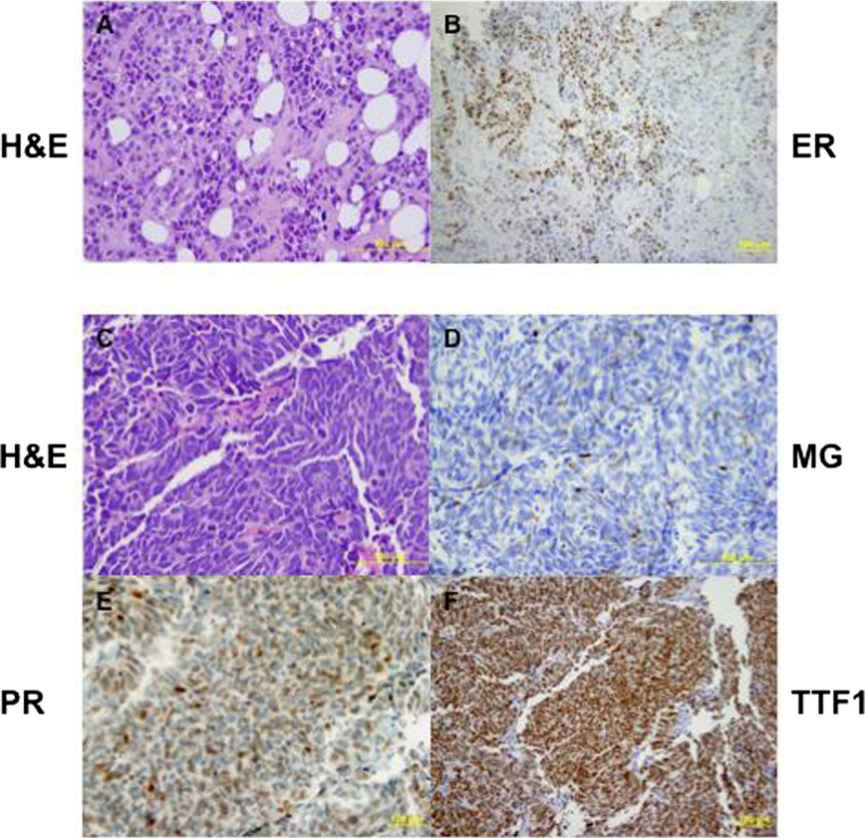
**Histology of original invasive (A-B) and recurrent (C-F) breast carcinoma.** H & E stained section showing invasive ductal carcinoma, grade 3 from 2005. **(B)** The carcinoma was positive for estrogen receptor (ER) and progesterone receptor (PR), and negative for Her2. **(C)** The recurrent small cell carcinoma from 2012 is characterized by spindle cells with frequent mitosis and single cell necrosis. **(D-F)** The recurrent carcinoma is focally positive for mammoglobin (MG), PR, and TTF1.

A 53-year-old Peruvian woman presented to our institution for a second opinion on a left axillary mass. She had never smoked, and had not been chronically exposed to cigarette smoke. She had no history of known significant toxic exposures. She was diagnosed with breast cancer at the age of 46, when she felt a left breast lump. Core biopsy of the 2 cm mass showed a grade 3 infiltrating ductal carcinoma with lymphovascular invasion. The tumor was estrogen receptor positive (ER+), progesterone receptor positive (PR+), and HER2-negative (Figure 1A-B). She received 6 cycles of neoadjuvant docetaxel, doxorubicin, and cyclophosphamide (TAC).

**Figure 2:**
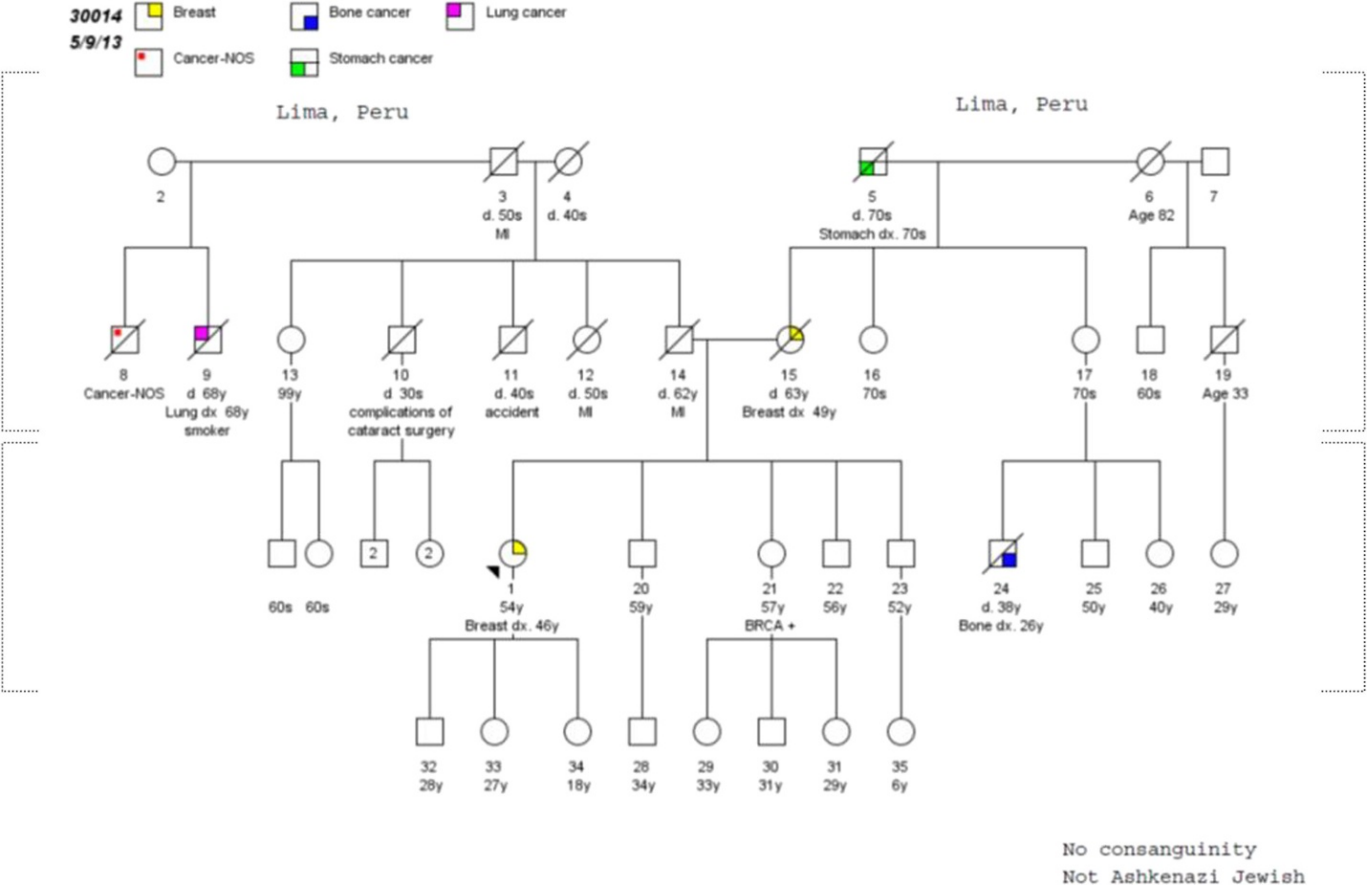
**Family pedigree.**

A four-generation pedigree (Figure 2) was positive for a history of breast cancer at age 49 years in her mother, and stomach cancer after age 70 years in her maternal grandfather. Sequencing of the *BRCA1* and *BRCA2* genes revealed a deleterious nonsense mutation in *BRCA2* (c.5374del4), which is predicted to result in a stop codon at amino acid 1723.

Following chemotherapy, the patient had bilateral mastectomy, which showed no residual invasive cancer. None of the seven sampled axillary lymph nodes contained carcinoma. She then received adjuvant tamoxifen for 1.5 years, until she had a bilateral oophorectomy. Afterwards, she started letrozole.

Three years later, while on letrozole, the patient felt a hard lump in her left axillary tail, measuring 2.5 × 3 cm. PET/CT scan showed only a focus of markedly increased activity in the lower left axilla, measuring 1.7 cm, with no SUV specified. Biopsy of this mass revealed a small cell carcinoma, with densely packed cells with scant cytoplasm and finely granular chromatin. Evaluation of biomarkers was as follows: ER-negative, PR-positive with 30% staining, HER2-negative. Ki-67 was 65%. The cells were strongly positive for CD56, chromogranin, synaptophysin, and TTF-1. They were also CK7+, CK20-, and weakly positive for mammoglobin. Upon comparison, the initial breast cancer was different both histologically and immunohistochemically from the small cell cancer. The original breast cancer was negative for TTF-1 and chromogranin.The left-sided mass was resected, and final surgical pathology showed a poorly-differentiated small cell neuroendocrine carcinoma (see Figure 1C-F), measuring 2.5 × 2.5 × 1.8 cm. The resection was followed by 4 cycles of adjuvant cisplatin and etoposide, as well as radiation therapy. The patient currently remains with no evidence of disease.

To compare the two tumors at the molecular level, we performed targeted Next-Generation Sequencing (NGS) with a panel that includes 2,855 cancer-associated mutations in 50 genes. The breast cancer specimen was positive for a heterozygous mutation in *PIK3CA* (c. 1624 G > A, p.E42K) and a heterozygous 13-basepair deletion in *TP53* (c.639-651del, p.H214fs). The small cell cancer specimen was positive for the same mutation in *PIK3CA*, but negative for the mutation in *TP53* despite adequate sequencing coverage for amplicons in that gene (>900X). Chromosomal microarray analysis (CMA) on the small cell specimen identified a heterozygous deletion of chromosome 17p, encompassing the TP53 locus. There was not enough tissue available from the original breast biopsy to perform CMA, so it is unclear if the original breast cancer had this 17p deletion.

Though it would be ideal to test the patient’s peripheral blood to definitively determine that the *PIK3CA* and *TP53* mutations are somatic, this is not possible because the patient has moved out of state. In the absence of such testing, we postulate that these two mutations are likely somatic because that specific *PIK3CA* mutation is identified in the COSMIC database as a somatic mutation, and it is known to be rather common, as described below. It has not been described as a germline mutation. The heterozygous 13 basepair deletion in TP53 is also likely somatic because it was not seen in the small cell cancer specimen, as would be expected if it were a germline mutation. Furthermore, we believe the *TP53* mutation is a pathogenic mutation, though it has not been described before, because it is a frameshift mutation.

Of note, both mutations also showed evidence of mosaicism, further supporting their likely somatic nature. The *PIK3CA* p.E42K mutation was present in 40.8% of the initial breast carcinoma, and in 25% of the small cell cancer specimen. Similarly, the *TP53* deletion was present in 35% of the initial breast cancer biopsy. In contrast, all the other mutations which were found were present in 98-100% of each sample, implying that they are more likely ubiquitous germline mutations. For example, on NGS, both samples also carried common germline variants in *SMARCB1* (c.1119-41G > A) and *PIK3CA* (c.1173A > G). These mutations are believed to be germline variants because they are both described in the NCBI SNP database, with the *SMARCB1* mutation occurring in 11% of people as a benign germline variant, and the *PIK3CA* mutation occurring in 7.3% of people.

Based on genetic analysis of the initial tumor and the second breast cancer, we suggest that the second cancer may have been a recurrence of the initial breast cancer several years before. Both tumors carried the same PIK3CA mutation, but only the initial breast cancer carried the p53 mutation. PIK3CA mutations have been reported in 18-45% of breast cancer cases [6]. Furthermore, the specific mutation that was identified in both tumors in our patient is common, comprising 18-26% of all *PIK3CA* mutations in some series [6, 7]. Most breast cancer recurrences retain their *PIK3CA* mutations, establishing it as a driver mutation [8].

On the other hand, the TP53 mutation that was identified in the breast cancer specimen was not present in the small cell cancer. We believe that one of two scenarios could explain this discrepancy. In the first scenario, the PIK3CA mutation was an early event, and one sub-clone developed an additional *TP53* mutation, while the clone that recurred as a small cell carcinoma did not. Alternatively, the starting clone may have contained both the *PIK3CA* and *TP53* mutations, but the sub-clone that recurred as a small cell carcinoma lost the mutant *TP53* allele with the documented deletion of 17p. Although we were not able to perform CMA on the breast cancer specimen, we favor the first scenario because the NGS read count shows more reads from the *TP53* locus in the small cell specimen, which is the opposite of what one would expect if there had been a loss of material at 17p in the small cell carcinoma.

Though we can never know with 100% certainty whether the two tumors are clonally related, we believe that there is a very good chance that this is the case because of the retained identical *PIK3CA* mutation. Furthermore, in the presence of highly error-prone DNA repair caused by a germline *BRCA2* mutation [9], we hypothesize that the clone from the original tumor underwent such a high degree of dysregulation that it took on a completely different pathology, morphology, and immunohistochemistry pattern. We propose this case history as an important example of the effects of therapeutic stress combined with host deficiencies in DNA repair. Other reports have shown that BRCA deficient tumors are very susceptible to certain types of therapeutic stress, such as poly (ADP-ribose) polymerase (PARP) inhibition. Many cells die, but others survive while exhibiting major aberrations such as chromatid breaks and tri-and quadri-radial chromosomes [10]. While TAC chemotherapy was very effective in eradicating the majority of this patient’s first cancer, the remaining portion might have been a very resistant one which was prone to such significant changes in the absence of adequate DNA repair. Therapeutic stress itself may be a vehicle of clonal selection and additional mutational events in cancer cells. To our knowledge, there have been no previous reports of small cell carcinoma of the breast occurring in a patient with a BRCA2 mutation.

Diagnostically and therapeutically, the implications of this case are quite intriguing. Clinically, when a patient has a second breast cancer, we review the biology, morphology, and histopathology to determine whether it may be a new primary or a recurrence of the previous cancer. Especially in patients who have dysregulated DNA repair, these characteristics of the cancer may not reveal the entire story. Some cancers which we label as “second primaries” could, in fact, be recurrent cancers. For example, recent evidence suggests that genomically, some breast tumors are more similar to certain ovarian cancers than other breast cancers [11].

## Conclusion

Our findings highlight the potential of longitudinal and serial profiling of tumors to study which events confer resistance or a survival advantage for tumor cells. It also demonstrates how therapy can eliminate dominant clones and select for rare but resistant ones. We demonstrate here clonal evolution of a tumor that recurred with a histologically distinct picture, but genomically carried the hallmarks of the original tumor. This could potentially have very important therapeutic implications, as better understanding of the host factors which affect the way the cells recover from DNA damage could help guide treatment options and allow us to more effectively harness therapeutic stress.

## Consent

Written informed consent was obtained from the patient for publication of this Case report and any accompanying images. A copy of the written consent is available for review by the Editor-in-Chief of this journal.

## Abbreviations

DCIS: Ductal carcinoma in Situ
CK: Cytokeratin
ER: Estrogen receptor
PR: Progesterone receptor
TAC: Docetaxel (Taxotere), adriamycin, cyclophosphamide chemotherapy regimen
NGS: Next generation sequencing
CMA: Chromosomal microarray analysis.
